# Cardiovascular risk factors in HIV infected individuals: Comparison with general adult control population in Greece

**DOI:** 10.1371/journal.pone.0230730

**Published:** 2020-03-30

**Authors:** Giota Touloumi, Natasa Kalpourtzi, Vasileios Papastamopoulos, Vasilios Paparizos, Georgios Adamis, Anastasia Antoniadou, Maria Chini, Argiro Karakosta, Konstantinos Makrilakis, Magda Gavana, Apostolos Vantarakis, Mina Psichogiou, Simeon Metallidis, Nikolaos V. Sipsas, Helen Sambatakou, Christos Hadjichristodoulou, Paraskevi V. Voulgari, George Chrysos, Charalambos Gogos, Grigoris Chlouverakis, Grigoris Tripsianis, Yannis Alamanos, George Stergiou, on behalf of the AMACS and EMENO

**Affiliations:** 1 Department of Hygiene, Epidemiology & Medical Statistics, Medical School, National & Kapodistrian University of Athens, Athens, Greece; 2 5th Department of Internal Medicine & Infectious Diseases Unit, Evaggelismos General Hospital, Athens, Greece; 3 AIDS Unit, Clinic of Venereologic & Dermatologic Diseases, Syngros Hospital, Athens, Greece; 4 1st Dept of Internal Medicine and Infectious Diseases, Gennimatas General Hospital, Athens, Greece; 5 4th Dept Of Internal Medicine, Attikon General Hospital, Medical School, National and Kapodistrian University of Athens, Athens, Greece; 6 3rd Dept Of Internal Medicine—Infectious Disease Unit, Red Cross General Hospital, Athens, Greece; 7 Hellenic Diabetes Association, Medical School, National & Kapodistrian University of Athens, Athens, Greece; 8 Lab of Primary Health Care, General Medicine & Health Services Research, Medical Department, School of Health Sciences, Aristotle University of Thessaloniki, Thessaloniki, Greece; 9 Environmental Health, Medical School, University of Patras, Patras, Greece; 10 1st Department of Internal Medicine, Medical School, National and Kapodistrian University of Athens, Athens, Greece; 11 1st Internal Medicine Department, Infectious Diseases Unit, Ahepa University Hospital, Aristotle University of Thessaloniki, Thessaloniki, Greece; 12 Infectious Diseases Unit, Department of Pathophysiology Laikon Athens General Hospital and Medical School, National and Kapodistrian University of Athens, Athens, Greece; 13 2nd Dept of Internal Medicine, HIV Unit, Hippokration General Hospital, Medical School, National and Kapodistrian University of Athens, Athens, Greece; 14 Department of Hygiene and Epidemiology, Medical Faculty, University of Thessaly, Larissa, Greece; 15 Department of Internal Medicine, Rheumatology Clinic, University of Ioannina, Ioannina, Greece; 16 Infectious Diseases Unit, Tzaneion General Hospital of Piraeus, Athens, Greece; 17 Dept of Internal Medicine & Infectious Diseases, Patras University General Hospital, Patras, Greece; 18 Division of Biostatistics, School of Medicine, University of Crete, Heraklion, Greece; 19 Department of Medical Statistics, Medical Faculty, Democritus University of Thrace, Alexandroupolis, Greece; 20 Institute of Epidemiology, Preventive Medicine and Public Health, Corfu, Greece; 21 Hypertension Center, STRIDE-7, Third department of Medicine, School of Medicine, National & Kapodistrian University of Athens, Sotiria Hospital, Athens, Greece; Harvard Medical School, UNITED STATES

## Abstract

**Background:**

Although combined antiretroviral therapy has substantially improved the prognosis of people living with HIV (PLHIV), mortality remains higher compared to the general population, mainly due to higher prevalence of non-HIV-related comorbidities, including cardiovascular diseases (CVD). We assessed the prevalence of CVD risk and its contributing factors in adult PLHIV versus general population controls in Greece.

**Settings:**

Cross-sectional comparison of PLHIV (Athens-Multicenter-AIDS-Cohort-Study; AMACS) versus general population controls (National health examination survey; EMENO).

**Methods:**

All HIV-infected adults with ≥1 measurement of interest (blood pressure, lipids, glucose, weight, height) between 2012–2014 and all EMENO participants (2014–2016) were included. Ten-year total CVD risk was estimated using the Framingham (FRS) or the Systematic Coronary Risk Evaluation (SCORE) equations.

**Results:**

5839 PLHIV (median age:41.6 years, 85.4% males) and 4820 controls (median age:48 years, 48.4% males) were included. Adjusting for age, sex and origin, PLHIV were more likely to be current smokers (adjusted OR:1.53 [95% CI:1.35–1.74]) and dyslipidemic (aOR:1.18; [1.04–1.34]), less likely to be obese (aOR:0.44 [0.38–0.52], with no differences in hypertension, diabetes or high (≥20%) FRS but with greater odds of high (≥5%) SCORE (aOR:1.55 [1.05–2.30]). Further adjustment for educational level, anti-HCV positivity and BMI showed higher prevalence of hypertension in PLHIV.

**Conclusions:**

Despite the relative absence of obesity, PLHIV have higher prevalence of traditional CVD risk factors and higher risk of fatal CVD compared to general population. Regular screening and early management of CVD risk factors in PLHIV should be of high priority for CVD prevention.

## Introduction

Mortality among people living with HIV (PLHIV) has decreased substantially since the introduction of combined antiretroviral therapy (cART). Mortality rates, however, remain higher in PLHIV compared to the general population [1–3]. The difference is mainly attributed to the higher prevalence of non-HIV related comorbidities, with cardiovascular diseases (CVD) being the most important contributor [4–7]. HIV itself, through chronic immune activation/inflammation, immune dysfunction or toxicities and metabolic complications caused by cART, has been associated with the premature development of chronic comorbidities in PLHIV [8–10]. Higher prevalence of modifiable CVD risk factors among HIV-infected as compared to HIV negative individuals could be another contributing factor [11,12].

Smoking, a modifiable risk factor that is strongly associated with CVD, is highly prevalent among PLHIV [13,14]; On the other hand, factors including body mass index (BMI), a mediator of CVD risk through its strong association with diabetes mellitus (DM), hypercholesterolemia, and hypertension [15], have been reported to be less prevalent in PLHIV compared to the general population in most [11,12] but not all studies [16]. Results from studies comparing hypertension and/or DM prevalence in PLHIV with the general population are contradictory [11,16–21]. Dyslipidemia is a well described side effect of older antiretrovirals, but new generation cART is less toxic with fewer metabolic complications [22,23]. In the new generation cART era, evaluating and understanding the differences in CVD risk factors’ prevalence, and particularly in the modifiable ones, between PLHIV and HIV-negative individuals has the potential to provide insights that could improve clinical management and benefit both physicians and patients.

The aim of this study was to estimate i) the prevalence of specific non-AIDS related comorbidities and established CVD risk factors (diabetes, dyslipidemia, hypertension, obesity, smoking), ii) the total CVD risk estimated using the 10-year Framingham risk score (FRS) or the European Systematic Coronary Risk Evaluation (SCORE) in adult PLHIV in comparison to general population controls in Greece. For this purpose, data from a large ongoing Greek cohort of HIV-infected individuals, were compared cross-sectionally with data from a recent health examination survey in a representative sample of the general adult population in Greece.

## Methods

### Data sources

Data for the HIV-infected individuals were derived from the “Athens Multicenter AIDS Cohort Study” (AMACS). AMACS is a collaborative, ongoing, population-based cohort study, initiated in 1996.Currently, 14 out of the 18main clinics that follow PLHIV in Greece, participate in the study. Data are provided by the clinics after de-identification. The study has been approved by the Athens University IRB (http://en.uoa.gr/), the HCIDC IRB (http://www.keelpno.gr/en-us/home.aspx) and the National Organization of Medicines (http://www.eof.gr/web/guest/home).

For data collection a standardized protocol is used. Data are extracted from patients’ files, updated regularly and thoroughly checked for errors and inconsistencies. For the current analysis data cut-off date was December 2014.

Data for the general population were derived from the health examination survey “National Survey of Morbidity and Risk Factors” (EMENO). EMENO is a population-based cross-sectional survey aiming to include 6000 adults aged ≥18 years. The study design has been described in detail elsewhere [24]. Briefly, multistage stratified random sampling based on 2011 census was applied to select the sample. During home visits, trained interviewers administered a standardized questionnaire to study participants and recorded all medications currently taken (name and dosage) by study participants whereas trained physicians collected blood samples, took automated blood pressure measurements and measured height and weight using standardized equipment and methodology. Total cholesterol, HDL-C, LDL-C, triglycerides, fasting plasma glucose and HbA1c were determined in all collected blood samples. EMENO fieldwork took place during May 2013-June 2016. All participants provided signed informed consent. The study was approved by the Athens University IRB (http://en.uoa.gr/) and the Hellenic Data Protection Authority (www.dpa.gr).

### Eligibility criteria

All AMACS participants who had at least one measurement of interest (blood pressure, cholesterol, glucose, triglycerides, HDL, weight, height) between 2012–2014 and aged ≥18 years at the selected visit, were included in the current study. If more than one visit was available within 2012–2014, the most recent was selected.

All EMENO participants who had at least one measurement of interest were included in the current study. HIV positive individuals were excluded from all analyses.

### Definitions

Hypertension was defined as systolic blood pressure ≥140 mmHg and/or diastolic blood pressure ≥90 mmHg, or taking antihypertensive medication [25].Diabetes was defined as fasting bloodglucose≥126 mg/dL (7.0 mmol/l) or taking antidiabetic treatment (including dietary intervention only). In EMENO, participants were also classified as diabetics if they had HbA1c≥6.5% (48 mmol/mol) to avoid excluding those who had not fasted for 8 hours during blood draw. In AMACS, physicians’ diagnosis of diabetes was also included [26].

Dyslipidemia was defined as elevated total cholesterol ≥240 mg/dl(6.21 mmol/l), and/or decreased high-density lipoprotein (HDL)-cholesterol ≤35mg/dl(0.91 mmol/l), and/or elevated triglycerides ≥200 mg/dl(5.17 mmol/l), or taking antilipidemic medications [27]. BMI was based on physicians’ measurements of height and weight;BMI≥30Kg/m^2^ was considered indicative of obesity. AMACS participants were classified as infected with hepatitis C virus (HCV) if they had a positive anti-HCV test or a detectable HCV-RNA in their medical records and as infected with hepatitis B virus (HBV) if they had a positive HBsAg. EMENO participants who provided signed informed consent had been screened using anti-HCV and HBsAg tests.

The 10-year total risk of fatal and non-fatal CVD was estimated using the Framingham risk score (FRS) [28] and the 10-year risk of fatal CVD using the Systematic Coronary Risk Evaluation (SCORE) [29]. Those with FRS≥20% and those with SCORE≥5% were considered as being at high risk. As SCORE can only be estimated in those aged>20 years, FRS and SCORE risk estimation were restricted to these ages. In addition, SCORE was estimated only in persons without diabetes.

### Statistical analysis

Sampling weights, being the reciprocal of the selection probabilities, multiplied with post-stratification weights based on 2011 census were applied to EMENO data to allow for differences in age and sex distribution between the final sample and the adult Greek population. To adjust for non-response, as a sub-sample of the interviewed individuals participated in the physical examination and provided blood samples, the inverse probability weighting method was applied. Weights were estimated through multivariable logistic regression.

In AMACS, as the number of participants with available measurements on CVD risk factors (blood pressure, cholesterol, glucose, triglycerides, HDL, BMI) varied by risk factor, we imputed the missing values using the multiple imputation method; data were imputed separately for men and women using joint modelling assuming multivariate normal distribution [30]. Partially observed variables included in the model were transmission category, systolic and diastolic blood pressure, total cholesterol, HDL, blood glucose, triglycerides, HBV and HCV coinfection, height, weight, educational level, smoking status, CD4 counts (in square root scale), HIV-RNA level (in log_10_ scale) and diagnoses of hypertension, diabetes or dyslipidemia. Completely observed variables included in the model were age, country of origin (Greek vs. other) and AIDS status. Ten imputed datasets were created using the jomo package [31] in R, with 2,000 burn-in iterations and 500 iterations between each imputation. For the main analysis, the imputed AMACS data were used.

Weighted multivariable logistic regression models, assigning a weight of one to AMACS participants, were used to evaluate the difference in prevalence of CVD risk factors and of high FRS or SCORE risk between the two groups. Weighted multivariable fractional logistic regression models [32] were applied to model FRS or SCORE 10-year risk. For the main analyses, all models were adjusted for age (as continuous variable, linear or quadratic function depending on the dependent variable (outcome) and on the goodness-of-fit test results), gender and origin (Greek/other; model 1).Models were additionally adjusted for presence of HCV or HBV infection and for educational level (primary, secondary, university or higher; model 2) and, in addition, for BMI (<25, 25–29.9, ≥30 Kg/m^2^;model 3).

Based on main modelling approach (i.e., adjusting for age, sex and origin) we estimated the predicted marginal probabilities of each investigated outcome and its 95% CI by HIV status [33,34]. Given the age, sex and country of origin distribution of the combined AMACS and EMENO population, these are the predicted probabilities of each outcome, if all were infected with HIV and vice-versa.

### Sensitivity analyses

A set of pre-specified sensitivity analyses were carried out. Firstly, we repeated the main analysis after excluding HBV or HCV positive persons from both populations; secondly, we carried out a complete-case analysis where all models mentioned above were fitted to the original data without imputing missing data in AMACS; and third, we applied a matched case-control design to compare the two populations: for each investigated outcome, HIV-infected individuals were one-to-one matched to individuals from the general population by age (±1 year), sex and origin. Models appropriate for individually matched case-control studies were applied.

All statistical analyses were performed using the statistical software STATA (version 13.0).

## Results

In total 6006 individuals were enrolled in the EMENO study, with overall response rate 72%. From the analysis, 13 with unknown age, and 2 who were found to be HIV+ were excluded. Of the remaining 5991 participants, 4820 had at least one measurement of the variables of interest (BP, cholesterol levels, triglycerides, fasting glucose, HbA1c, weight, height). The 1171 excluded patients were more likely to be from urban areas and the youngest age group (i.e., 18–29 years old) and less likely to be unemployed, have a chronic disease, have children and of Greek origin. A logistic regression model adjusted for all these factors was fitted to estimate the probability of response.

In AMACS, 6880 HIV-infected adults had at least one clinic visit during 2012–2014; among them, 5839 had available at least one measurement of the variables of interest. Thus, the total sample size consisted of 5839 HIV-infected and 4820 controls from the general population. Baseline characteristics of the two populations and HIV-specific characteristics are presented in Table 1. The majority of the AMACS patients were men (85.4%), infected through sex between men (55.6%), with median (IQR) age 41.6 (34.0–49.7) years. For the vast majority of the AMACS patients, their last available clinic visit within 2012–2014 was in 2014 (82%). By then, they had been infected for a median of 6.6 years and 14% of them had developed clinical AIDS. The majority of the HIV-infected patients (86.1%) were on ART and 74.7% had HIV-RNA below 50 copies/ml.

**Table 1:** Descriptive characteristics of the study population by HIV status.

	HIV-infected (N = 5839)	General population (N = 4820)	p-value
	N (%)	N (weighted %)	
Gender			<0.001
*Male*	4984 (85.4)	2063 (48.4)	
*Female*	855 (14.6)	2757 (51.6)	
Age group (years)			<0.001
*18–39*	2604 (44.6)	1099 (35.9)	
*40–49*	1769 (30.3)	846 (17.8)	
*50–59*	973 (16.7)	931 (15.7)	
*60–69*	358 (6.1)	926 (12.8)	
*70+*	135 (2.3)	1018 (17.9)	
Country of origin			<0.001
*Greece*	5014 (85.9)	4561 (94.4)	
Education			<0.001
*Up to Primary*	139 (2.4)	1774 (31.9)	
*Secondary*	1315 (22.6)	2043 (46.0)	
*University or higher*	967 (16.6)	926 (19.4)	
*Unknown*	3418 (58.5)	77 (2.7)	
	N = 5528	N = 4240	
HBsAg positive	314 (5.7)	72 (1.9)	<0.001
	N = 5223	N = 4242	
Anti-HCV positive	726 (13.2)	34 (0.8)	<0.001
	N = 5186	N = 3792	
Injecting Drug Use	587 (11.3)	24 (0.9)	<0.001
Mode of HIV-Infection^a^			
*MSM*	3246 (55.6)	-	
*PWID*	587 (10.1)	-	
*MSW*	1334 (22.9)	-	
*Other /Unknown*	672 (11.5)	-	
Year of baseline visit			
*2012*	218 (3.7)	-	
*2013*	832 (14.3)	-	
*2014*	4789 (82.0)	-	
AIDS at baseline	816 (14.0)	-	
Not on ART at baseline	814 (13.9)	-	
ART type^b^			
*NNRTI*	1988 (34.1)	-	
*INSTI*	835 (14.3)	-	
*Boosted PI*	2174 (37.2)	-	
*Other*	28 (0.5)	-	
Median (IQR) CD4 counts (cells/μl)	606 (416, 819)	-	
Years since diagnosed Median (IQR)	6.6 (2.8, 13.2)	-	
Years since ART initiation Median (IQR)	6.4 (2.9, 13.3)	-	
VL <50 copies/ml (N = 5737) N (%)	4286 (74.7)	-	

^a^: MSM: Men having Sex with Men, PWID: People Who Inject Drugs, MSW: Men having Sex with Women;

^b^: ART: Anti-Retroviral Therapy, NNRTI: Non-Nucleoside Reverse Transcriptase Inhibitors, INSTI: Integrase Strand Transfer Inhibitors, Boosted PI: Boosted Protease inhibitors.

Sex distribution was balanced in the general population (48.4% male), which presented a higher median age than the AMACS population [median (IQR) age: 48.0 (34.9–64.0) years]. Most participants in both populations were of Greek/Western Europe (WE) origin, although a larger percentage in the AMACS cohort was from other regions. HBV or HCV co-infection was significantly more prevalent among HIV-infected individuals compared to the general population.

Based on complete cases data, crude (unadjusted) prevalence of hypertension, diabetes, obesity and high FRS or SCORE were significantly higher in the control group. On the contrary, prevalence of dyslipidemia and current smoking were significantly higher in HIV-infected individuals compared to the general population (Table 2). However, the number of individuals with available data on CVD risk factors varied substantially in the AMACS cohort, being smallest for hypertension and obesity, whereas it was relatively consistent in the EMENO (Table 2). Among AMACS participants, hypertension data was more likely to be missing in women, younger individuals, people who inject drugs (PWID), and in persons without diabetes, subgroups that are associated with lower rates of hypertension; BMI data was more likely to be missing in those reported to be infected through sex between men and women, and in older individuals, who are, in general, individuals with higher BMI, and less likely in PWID; thus, the prevalence estimates of hypertension and of high FRS were lower, and those of obesity, higher, after multiple imputation. However, results from crude comparisons between the two groups, using the imputed data, were similar to those from complete cases data.

**Table 2:** Number of people with available data and crude prevalence (%) of cardiovascular disease (CVD) risk factors and of risk of CVD by population.

		HIV-Infected (N = 5839)		General population (N = 4820)		p-value
	N	Crude prevalence (95% CI)	Crude Prevalence (95% CI) after MI^a^	N	Crude weighted Prevalence (95% CI)	
Hypertension	2057	35.6 (33.6, 37.7)	34.4 (32.6, 36.3)	4751	41.1 (39.3, 43.0)	<0.001
Diabetes	5703	7.2 (6.5, 7.9)	7.2 (6.6, 7.9)	4391	11.3 (10.2, 12.5)	<0.001
Dyslipidemia	5661	48.6 (47.3, 49.9)	48.7 (47.4, 50.0)	4419	44.1 (42.1, 46.1)	<0.001
Current smoking	3047	58.5 (56.7, 60.2)	59.1 (57.6, 60.6)	4711	38.7 (36.7, 40.7)	<0.001
Obesity	2143	10.6 (9.4, 12.0)	15.8 (14.6, 17.1)	4761	33.4 (31.6, 35.2)	<0.001
High FRS^b^	1266	20.1 (18.0, 22.4)	16.6 (15.5, 17.8)	4145	27.3 (25.6, 29.0)	<0.001
FRS^b^ (Mean, 95% CI)	1266	12.3 (11.6, 13.1)	11.2 (10.9, 11.6)	4145	15.5 (14.8, 16.2)	<0.001
High SCORE^b^	1158	5.4 (4.2, 6.8)	5.4 (4.7, 6.2)	3577	17.3 (15.9, 18.8)	<0.001
SCORE^b^ (Mean, 95%CI)	1158	1.2 (1.0, 1.3)	1.2 (1.1, 1.3)	3577	2.8 (2.6, 3.0)	<0.001

^a^: Crude prevalence estimated after filling in missing data in the AMACS patients using the multiple imputation (MI) method.

^b^: Framingham risk score (FRS) used to estimate the 10-year risk of fatal and non-fatal CVD and the Systematic Coronary Risk Evaluation (SCORE) to estimate the 10-year risk of fatal CVD. FRS risk ≥20% (High FRS) and SCORE risk score ≥5% (High SCORE) were considered as high risk. FRS was estimated for those aged>20 years and SCORE for non-diabetics aged >20years.

**Fig 1:**
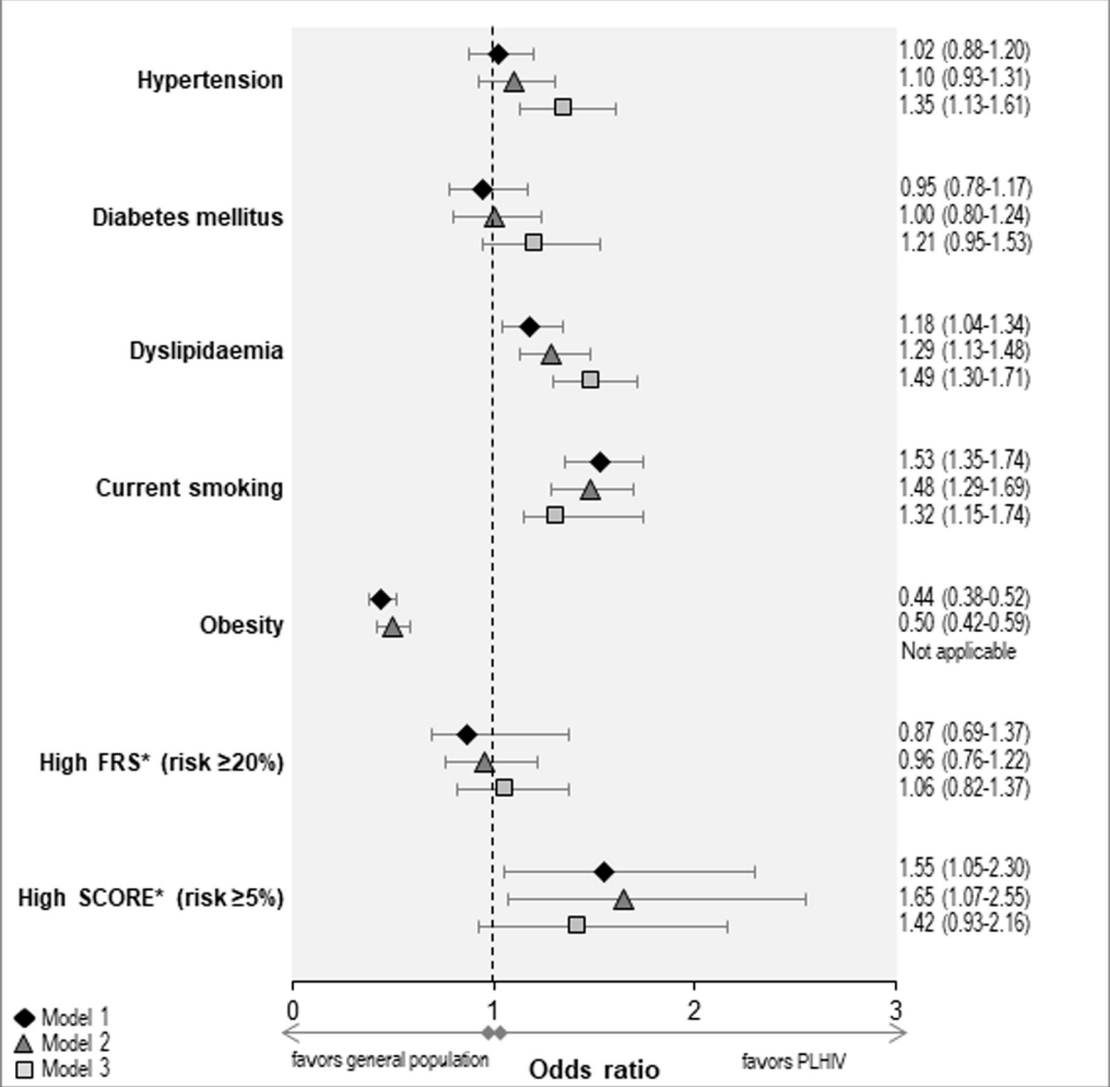
Adjusted odds ratio (OR) and 95% confidence interval (95% CI) of cardiovascular (CVD) risk factors and total CVD risk comparing HIV-infected adults to the general population. Model 1: adjusted for age, sex and country of origin; Model 2: Model 1 plus additional adjustment for HBsAg(+) or anti-HCV(+) and educational level; Model 3: Model 2 plus additional adjustment for body mass index (BMI). Ten-year risk of fatal and non-fatal CVD using the Framingham risk score (FRS) was calculated for the individuals >20 years whereas 10-year risk of fatal CVD using the Systematic Coronary Risk Evaluation (SCORE) for the non-diabetic individuals >20 years. FRS risk ≥20% or SCORE risk score ≥5% were considered as high risk.

Adjusted for age, sex and origin, HIV-infected patients were more likely to be current smokers, dyslipidemic, and have a high SCORE, and less likely to be obese; there were no significant differences between the two groups in the odds of hypertension, diabetes or high FRS (Fig 1, model 1). Further adjustment for presence of HCV or HBV and for educational level did not alter substantially the main results (Fig 1, model 2). However, further adjusting for BMI, resulted in significant difference between the two groups in the odds of hypertension, which was higher in HIV-infected individuals (P = 0.001, Fig 1, model 3), and there was a trend towards higher odds of diabetes in HIV-infected individuals although the difference did not reach the nominal significance level (P = 0.112); the difference in the odds of dyslipidemia became more prominent while that of current smokers became less prominent (although still highly significant; P<0.001), as did for high SCORE (with the difference becoming marginally not significant; P = 0.105). Regarding the remaining covariates (data not shown), for most outcomes, the presence of HBV or HCV was not a significant predictor but the number of people from the general population with positive HBsAg and/or Anti-HCV was relatively small; the higher the educational level the lower the odds of all investigated outcomes; higher BMI was associated with higher odds of all investigated outcomes but smoking.

The predicted marginal prevalence of comorbidities and risk factors adjusted for age, sex and origin are presented in Table 3. FRS risk, modelled as a continuous variable and restricting the analysis to adults aged >20 years, did not differ significantly between the two groups. On the contrary, SCORE risk, modelled as a continuous variable and restricting the analysis to non-diabetic adults aged>20 years, was significantly higher in the HIV-infected.

**Table 3:** Predicted marginal prevalence (for the model that adjusts for age, sex and origin) and 95% confidence interval (CI) of comorbidities and risk factors by HIV status.

	HIV-Infected Predicted % (95% CI)	General Population Predicted % (95% CI)	p-value
Hypertension	38.0 (36.3, 39.8)	37.6 (35.7, 39.6)	0.771
Diabetes	9.0 (8.1, 9.9)	9.4 (8.4, 10.4)	0.648
Dyslipidemia	48.3 (46.9, 49.7)	44.5 (42.3, 46.7)	0.010
Current smoking	53.5 (51.9, 55.2)	43.8 (41.6, 46.0)	<0.001
Obesity	17.3 (15.8, 18.7)	31.4 (29.4, 33.4)	<0.001
High FRS^a^	21.1 (19.9, 22.2)	22.2 (21.1, 23.4)	0.195
10-year FRS^a^	13.2 (12.8, 13.5)	13.3 (12.9, 13.7)	0.606
High SCORE^a^	11.7 (10.8, 12.5)	10.4 (9.9, 11.0)	0.027
10-year SCORE^a^	2.2 (2.0, 2.3)	1.8 (1.8, 1.9)	<0.001

^a^: Framingham risk score (FRS) used to estimate 10-year risk of fatal and non-fatal CVD and Systematic Coronary Risk Evaluation (SCORE) to estimate 10-year risk of fatal CVD. FRS risk ≥20% (High FRS) and SCORE risk score ≥5% (High SCORE) were considered as high risk. FRS was estimated for those aged>20 years and SCORE for non-diabetics aged >20years.

Results from the sensitivity analyses were in line with those of the main analysis although significance levels were different due to the different number of observations contributing in each sensitivity analysis (Table 4).

**Table 4:** Adjusted odds ratio (OR) and 95% confidence interval (95% CI) of cardiovascular (CVD) risk factors and total CVD risk, comparing HIV-infected to the general population.

	Model	Complete Case analysis OR (95% CI)	Matched Case-Control analysis OR (95% CI)	Complete Case analysis excluding HBsAg(+) or anti-HCV(+) OR (95% CI)
Hypertension	1	0.99 (0.84, 1.17)	0.99 (0.84, 1.17)	1.05 (0.88, 1.25)
	2	1.35 (1.09, 1.67)	1.51 (1.14, 1.98)	1.38 (1.07, 1.08)
	3	1.31 (1.01, 1.08)	1.17 (0.78, 1.74)	1.35 (1.03, 1.79)
Diabetes	1	0.95 (1.15, 1.25)	0.97 (0.80, 1.18)	0.91 (0.74, 1.12)
	2	1.03 (0.80, 1.32)	0.98 (0.70, 1.37)	1.02 (0.79, 1.31)
	3	1.47 (1.05, 2.05)	1.17 (0.71, 1.92)	1.38 (0.98, 1.94)
Dyslipidemia	1	1.17 (1.03, 1.33)	1.14 (1.01, 1.29)	1.19 (1.04, 1.35)
	2	1.23 (1.06, 1.43)	1.08 (0.88, 1.33)	1.21 (1.04, 1.41)
	3	1.55 (1.29, 1.86)	1.43 (1.05, 1.94)	1.49 (1.23, 1.80)
Current smoking	1	1.42 (1.24, 1.63)	1.53 (1.33, 1.77)	1.30 (1.31, 1.50)
	2	1.31 (1.11, 1.55)	1.44 (1.14, 1.80)	1.30 (1.10, 1.53)
	3	1.25 (1.00, 1.56)	1.41 (1.00, 1.97)	1.23 (0.98, 1.53)
Obesity	1	0.28 (0.24, 0.34)	0.30 (0.24, 0.37)	0.30 (0.25, 0.37)
	2	0.31 (0.22, 0.43)	0.33 (0.21, 0.52)	0.34 (0.25, 0.48)
High FRS^a^ (age>20 years)	1	1.13 (0.87, 1.48)	1.08 (0.82, 1.43)	1.07 (0.81, 1.41)
	2	1.20 (0.84, 1.71)	1.20 (0.78, 1.85)	1.18 (0.81, 1.72)
	3	1.35 (0.86, 2.12)	1.28 (0.70, 2.34)	1.26 (0.79, 2.02)
High SCORE^a^ (age>20 years, non-diabetics)	1	1.39 (0.85, 2.29)	1.36 (0.79, 2.36)	1.28 (0.77, 2.13)
	2	1.55 (0.81, 2.95)	1.32 (0.56, 3.11)	1.40 (0.71, 2.74)
	3	1.37 (0.60, 3.12)	1.07 (0.29, 4.00)	1.31 (0.56, 3.07)

Model 1: adjusted for age, sex and origin; Model 2: additional adjustment for HBsAg(+) or anti-HCV(+) and educational level; Model 3: additional adjustment for Body Mass Index (BMI):Results from Sensitivity analyses.

^a^: Framingham risk score (FRS) used to estimate 10-year risk of fatal and non-fatal CVD and Systematic Coronary Risk Evaluation (SCORE) to estimate 10-year risk of fatal CVD. FRS risk ≥20% (High FRS) and SCORE risk score ≥5% (High SCORE) were considered as high risk.

## Discussion

In this work, we cross-sectionally compared the prevalence of CVD risk factors and the estimated total CVD risk between AMACS and EMENO participants. AMACS includes the majority of the HIV-infected adults living in Greece, and it is representative of the PLHIV on care [35]. EMENO consists of a representative sample of the adult general population living in Greece [24]. After adjusting for age, sex and country of origin, PLHIV in this study were more likely to be current smokers and dyslipidemic and had higher risk of fatal CVD, despite the fact that they were less likely to be obese; hypertension, diabetes, and FRS did not differ by HIV infection status. Further adjustment for educational level, anti-HCV positivity and BMI, in addition to dyslipidemia and smoking led to a significant difference in the prevalence of hypertension, with PLHIV having higher prevalence.

Smoking constitutes one of the strongest modifiable CVD risk factors. In this study, current smoking was high in both the general (weighted prevalence: 38.7%) and the HIV-infected population (crude prevalence: 59.1%), but still significantly higher in the latter. Most previous studies have reported higher frequency of current smoking in PLHIV [13,14] with prevalence ranging from 40% to 70% [36,37]. Social conditions, polysubstance abuse and psychiatric disorders may contribute to the higher prevalence in PLHIV [37]. The detrimental effects of smoking seem to be more pronounced in PLHIV, as smoking has been associated with higher risk of myocardial infraction in PLHIV than in population controls [38]. Thus, managing the factors contributing to the higher rates of smoking in PLHIV and encouraging smoking cessation is urgently needed.

In line with our results, most previous studies reported lower BMI in PLHIV than in HIV-uninfected counterparts [11,12]. Several studies have compared the prevalence of hypertension between PLHIV and HIV-uninfected individuals, but results are contradictory with some studies reporting higher [11,12,39], some comparable [20,19,40] and others lower [22,41] hypertension prevalence in PLHIV. In our study, the prevalence of hypertension adjusted for age, sex and country of origin was similar in PLHIV and in the general population. However, after further adjustment for BMI, a strong predictor of hypertension [42], it becomes evident that in fact, PLHIV have higher prevalence of hypertension, compared to the general population.

The effect of HIV infection or cART on dyslipidemia is well recognized [9,22,23]. In line with previous reports, we found that, adjusted for demographics, prevalence of dyslipidemia was higher in PLHIV than in the general population. This difference becomes more prominent after further adjustment for BMI. Whether the prevalence of diabetes is higher in PLHIV than in the general population is unclear as some studies have reported higher prevalence in PLHIV [16,18,21] whereas other failed to show it [11,17]. These studies though differed in the average age of the PLHIV, obesity prevalence and/or diabetes prevalence in the control group. In our study, HIV-infected individuals were relatively young (median age 41.6years), with low obesity prevalence, whereas diabetes prevalence in the control group was quite high (11.3%). In this setting, after adjustment only for demographics, prevalence of diabetes did not differ significantly between PLHIV and the general population. However, adjusting for BMI increased the difference between the two groups, suggesting higher prevalence in PLHIV (adjusted OR = 1.21 for HIV-infected vs general population) albeit to a non-significant level (P = 0.112). This finding was indicative that diabetes may appear in PLHIV even in the absence of obesity, a finding also previously reported [16]. Due to the lower body weight of PLHIV, healthcare professionals may not screen as often for diabetes, potentially resulting in undiagnosed diabetes.

As traditional risk factors are quite prevalent in PLHIV, accurate CVD risk stratification is very important. FRS and the European SCORE are two commonly used models for risk stratification. Other models, like the D:A:D (Data Collection on Adverse Events of Anti-HIV Drugs) equations, have also been developed to incorporate HIV-specific variables [43]. However, the best model to be used in clinical practice for PLHIV has not yet been identified [39,41].

In this study we estimated and compared FRS and SCORE CVD risk estimation between HIV-infected and the general population, but the results of the two score equations were not compatible. The prevalence of high FRS (≥ 20%) or the 10-year FRS risk score did not differ between the two groups in the basic model adjusted for demographics. On the contrary, the prevalence of high SCORE (≥5%) or10-year SCORE risk was significantly higher in HIV-infected people. Previous studies have reported contradictory results when comparing FRS risk in HIV-infected and uninfected individuals, with some reporting higher [44], some similar [22,45] and some even lower [46] risk in HIV-infected. It has been shown that the FRS equations underestimate CVD risk in PLHIV [35,47]. SCORE and FRS have been rarely compared in PLHIV. In a study it was found that SCORE and D:A:D equations were superior to FRS in estimating CVD risk in PLHIV[48]but, in another study conducted in HIV-infected individuals in the USA, it was found that FRS accurately estimated risk of CVD events, D:A:D underestimated risk whereas SCORE performed poorly [49]. However, SCORE equations have been developed and validated in European cohorts and thus they may be more appropriate for using in a European country. In any case, further research on validating and comparing the various CVD risk models in PLHIV is needed.

Our study has some limitations. In EMENO, standardized equipment and operating procedures were used across the country to evaluate CVD risk factors. In AMACS, data were extracted from patients’ files; some risk factors, anti-hypertensive treatment, diabetes and anti-diabetic treatment, may have been missing or recorded incorrectly. This would make harder to reveal the true differences between the two compared groups, with most probable underestimation of CVD risk factors in PLHIV. In AMACS, hypertension, obesity and to a lesser extent BMI had several missing values (often not measured, or measurement not recorded). We applied the MI method to impute missing data, but its validity depends on the accuracy of the imputing model. To increase the accuracy, we used many variables (auxiliary ones included) in the imputation model. In addition, a set of sensitivity analyses were carried out and all obtained results were in line with those of the main analysis, albeit with varying significance levels due to the different number of observations contributing in each analysis. AMACS and EMENO participants differed in the age, sex and, to a lesser extent, country of origin distribution. All analyses were adjusted for these factors. We also adjusted for other important variables, such as educational level (an indicator of socio-economic status) and anti-HCV positivity. However, we did not have data on poverty, diet and other behavioural variables. Thus, confounding of other unmeasured variables cannot be excluded in such cross-sectional observational studies.

## Conclusions

We estimated and compared the prevalence of hypertension, dyslipidemia, diabetes, current smoking, obesity and total CVD risk calculated by FRS or SCORE equations in adult PLHIV and in the general adult population living in Greece. Our results showed that, although PLHIV are less likely to be obese, they are more likely to be dyslipidemic, current smokers and to have high SCORE CVD risk. There is evidence that, for a given BMI, PLHIV are more likely to be hypertensive and, possibly even, diabetics. Health care providers should regularly screen HIV-infected persons for established CVD risk factors, even in the absence of obesity; smoking cessation campaigns are very important in this group. Assessment and management of established CVD risk factors are of high priority for HIV-infected patients’ care, potentially leading to improved health outcomes.
